# Cartilage Oligomeric Matrix Protein Gene Multilayers Inhibit Osteogenic Differentiation and Promote Chondrogenic Differentiation of Mesenchymal Stem Cells

**DOI:** 10.3390/ijms151120117

**Published:** 2014-11-05

**Authors:** Peng Guo, Zhong-Li Shi, An Liu, Tiao Lin, Fang-Gang Bi, Ming-Min Shi, Shi-Gui Yan

**Affiliations:** Department of Orthopaedic Surgery, the Second Affiliated Hospital, Medical College of Zhejiang University, NO. 88 Jiefang Road, Hangzhou 310009, China; E-Mails: 21218130@zju.edu.cn (P.G.); zlshi78@gmail.com (Z.-L.S.); liuan2013zju@gmail.com (A.L.); lintiao112009@gmail.com (T.L.); fanggangbi@gmail.com (F.-G.B.); shimingmin@gmail.com (M.-M.S.)

**Keywords:** cartilage oligomeric matrix protein, layer-by-layer self-assembly, gene delivery, mesenchymal stem cells, cell differentiation

## Abstract

There are still many challenges to acquire the optimal integration of biomedical materials with the surrounding tissues. Gene coatings on the surface of biomaterials may offer an effective approach to solve the problem. In order to investigate the gene multilayers mediated differentiation of mesenchymal stem cells (MSCs), gene functionalized films of hyaluronic acid (HA) and lipid-DNA complex (LDc) encoding cartilage oligomeric matrix protein (COMP) were constructed in this study via the layer-by-layer self-assembly technique. Characterizations of the HA/DNA multilayered films indicated the successful build-up process. Cells could be directly transfected by gene films and a higher expression could be obtained with the increasing bilayer number. The multilayered films were stable for a long period and DNA could be easily released in an enzymatic condition. Real-time polymerase chain reaction (RT-PCR) assay presented significantly higher (*p* < 0.01) COMP expression of MSCs cultured with HA/COMP multilayered films. Compared with control groups, the osteogenic gene expression levels of MSCs with HA/COMP multilayered films were down-regulated while the chondrogenic gene expression levels were up-regulated. Similarly, the alkaline phosphatase (ALP) staining and Alizarin red S staining of MSCs with HA/COMP films were weakened while the alcian blue staining was enhanced. These results demonstrated that HA/COMP multilayered films could inhibit osteogenic differentiation and promote chondrogenic differentiation of MSCs, which might provide new insight for physiological ligament-bone healing.

## 1. Introduction

Biomedical materials as medical devices have drawn great attention in both basic research and clinical application fields [1]. Surface modification of biomaterials (such as immobilization of the bioactive agents and chemical components), which can influence the manifestation and behavior of cells, has been demonstrated to be an efficient way to acquire a new nature for specific requirements [2,3,4,5]. In spite of this, the interface between implant and tissue is of great concern. Many efforts have been made to improve the integration of biomedical plants with the surrounding tissues which is vital for the success of various surgical procedures [6,7]. Considering the long-term efficiency of gene therapy, surface-mediated gene delivery is a promising method to promote the tissue ingrowth of the interface [8,9,10,11].

As first described by Decher *et al.* [12,13], the layer-by-layer (LbL) self-assembly technique has been widely used to construct ultrathin multilayers for gene transfection. The major advantage of the LbL technique is that it provides an approach to obtain controlled and sustaining release of DNA [14,15,16]. Although many studies [17,18,19] have focused on the osteogenic differentiation effects of multifarious gene films on mesenchymal stem cells (MSCs), there is still a lack of research on investigating the interactions for chondrogenic effects of gene films on MSCs.

A layer of calcified fibrocartilage is reported to be a major transition structure in the junction between bone and ligament [20]. This means it is important to sustain the cartilage layer in the interface between bone and ligament for physiological ligament-bone healing. Cartilage oligomeric matrix protein (COMP), being one of the most significant non-collagenous proteins in fibrous cartilage, belongs to the thrombospondin family and plays a vital role in the adhesion, proliferation and differentiation of cells [21,22,23]. Du YY *et al.* [24] found that COMP inhibited vascular smooth muscle cells calcification by interacting with bone morphogenetic protein 2 (BMP2). All these show that COMP has an important effect on cell differentiation.

Therefore, hyaluronic acid (HA)/COMP (lipid-DNA complex, LDc) multilayered films were constructed in the present study according to the HA/LDc fabrication method described before [15,25]. HA was chosen as a polyanion to interact with COMP-LDc because of its ideal biocompatibility and wide distribution in tissues. Multilayered films were presented as (HA/DNA)_n_ (*n* is the number of bilayers).

The object of this study was to construct and characterize the multilayered films functionalized with *pIRES-hrGFP-COMP* gene via the LbL technique, and to investigate whether the gene-functionalized films possessed biological activity, as well as to determine what effect these gene films had on the differentiation of MSCs *in vitro*.

## 2. Results and Discussion

### 2.1. Characterization of the Multilayer Films

#### 2.1.1. Contact Angle Measurement

**Figure 1:**
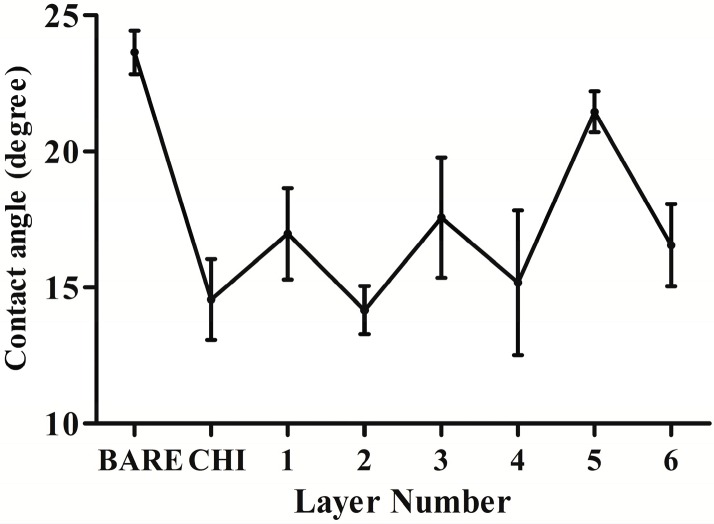
Water contact angles as a function of the number of coating layers. The even layer numbers correspond to DNA layers, and the odd layer numbers correspond to hyaluronic acid (HA) layers (*n* = 9).

Contact angle measurement shows the wettability of the modified substrates, dependent on the hydrophilic properties of the outermost layers. A larger contact angle generally indicates a better hydrophilicity. The contact angle of the primal layers decreased compared with the bare substrates, which suggested improvement of hydrophilicity when the substrates were coated with chitosan (CHI) (Figure 1). Also the contact angle of the following layers undulated from 30° to 10° as alternate deposit of the HA and DNA layers. The fluctuant changes of the contact angle indicated alterations of the outermost layers, which confirmed alternate fabrication of the HA and DNA layers on the substrates.

#### 2.1.2. UV–Vis Absorption

**Figure 2:**
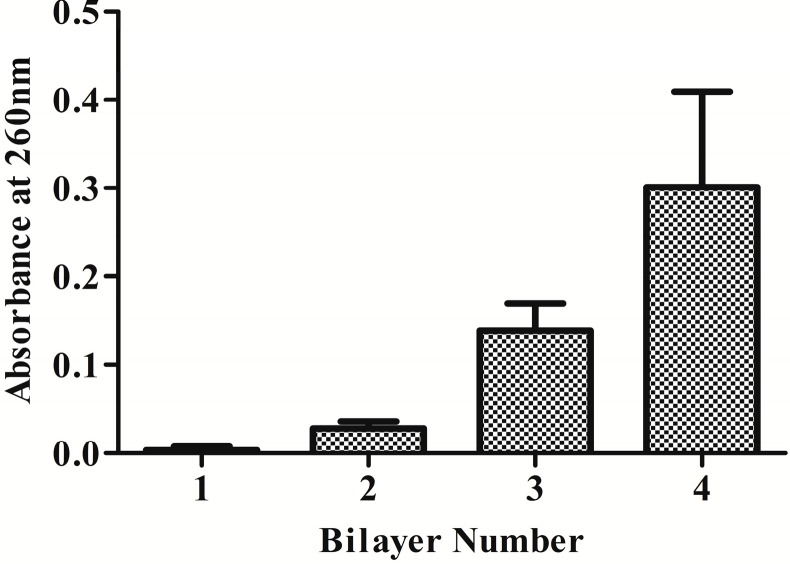
UV–vis absorbance of multilayered films at 260 nm as a function of layer number (*n* = 6).

UV–vis absorption at 260 nm is specifically contributed by the quantity of DNA, which thereby is a convenient way to monitor the build-up process by UV–vis spectrophotometer. The results of UV–vis absorbance at 260 nm of the multilayered DNA films are shown in Figure 2. The continuous increase of the UV–vis absorbance with the growth of bilayer number indicated that DNA was sequentially incorporated into the films, which also meant a successful fabrication process.

#### 2.1.3. Surface Morphology

**Figure 3:**
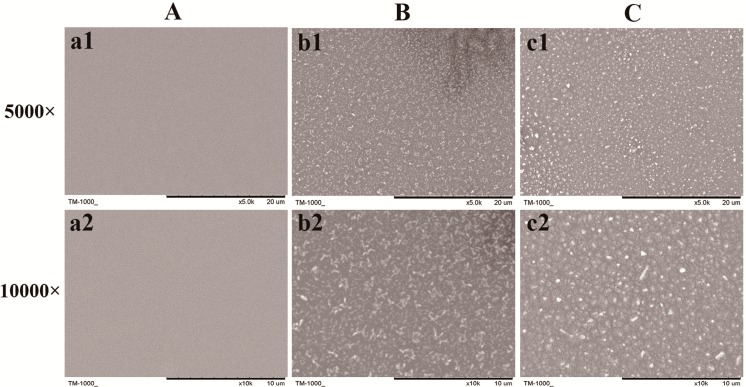
Scanning electron microscopy (SEM) images of the bare surface (**A**), (HA/DNA)_4_ films (**B**) and (HA/DNA)_8_ films (**C**); (**a1**), (**b1**) and (**c1**): 5000×; (**a2**), (**b2**) and (**c2**): 10,000×.

The morphology of the bare silicon surfaces, (HA/DNA)_4_ and (HA/DNA)_8_ films under different magnification was characterized by scanning electron microscopy (SEM). The multilayered films displayed a quite rough facade, which was different from the smooth and uniform appearance on the bare surface (Figure 3). (HA/DNA)_4_ films exhibited loose fragmental surfaces while (HA/DNA)_8_ films showed compact particle-like microstructures.

#### 2.1.4. Stability Assay and Enzymatic Degradation of Multilayered Films

**Figure 4:**
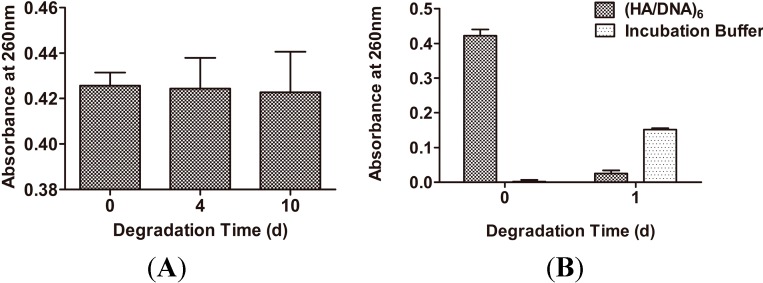
UV–vis absorbance of (HA/DNA)_6_ films and incubation buffer at 260 nm as function of time after incubation in (**A**) PBS at 37 °C, pH 7.4 (*n* = 3) and (**B**) the trypsin solution (0.1 mg/mL in PBS buffer) at 37 °C, pH 7.4 (*n* = 3).

PBS buffer was chosen as the model of the physiological condition for the stability of (HA/DNA)_6_ films. As shown in Figure 4A, there was hardly any decrease of the amount of DNA in the films even after 10 days incubation. This result suggested that the films were stable in the hypothetic physiological situation for more than 10 days.

However, it was quite different when multilayered films were immersed in the trypsin solution. The DNA was released from (HA/DNA)_6_ films to the incubation buffer, leading to a decrease of absorbance of the films and a remarkable increase of absorbance of the incubation buffer at day one (Figure 4B). This demonstrated that the enzymatic condition promoted the degradation process of multilayered films.

### 2.2. In Vitro Gene Transfection

**Figure 5:**
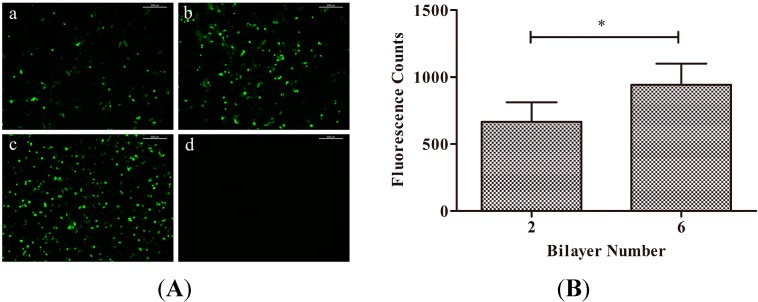
(**A**) GFP expression of 293T cells on different substrates after three days of culture. (**a**) (HA/DNA)_2_ films; (**b**) (HA/DNA)_6_ films; (**c**) tissue culture plates (TCPs) with Lipofectamine™ LTX and PLUS reagent, (**d**) TCPs. Scale bar: 200 μm; (**B**) The fluorescence counts of GFP expression in HEK293T cells after culturing on (HA/DNA)_2_ and (HA/DNA)_6_ films for three days (*n* = 5, mean ± SD, * *p* < 0.05).

Figure 5 shows the typical images of the expression of GFP by HEK293T cells cultured on different substrates after three days transfection. Cells on tissue culture plates (TCPs) without transfection were used as negative control, while TCPs transfected with Lipofectamine™ LTX and PLUS reagent were used as positive control. The GFP expression indicated efficient transfection by the DNA films. Also the expression of GFP on (HA/DNA)_6_ films was significantly higher (*p* < 0.05) than those on (HA/DNA)_2_ according to the different fluorescence counts (Figure 5B). The results suggested that increasing the layer number would contribute to higher gene transfection efficiency after three days of culture.

### 2.3. Cartilage Oligomeric Matrix Protein (COMP) Expression Detection

**Figure 6:**
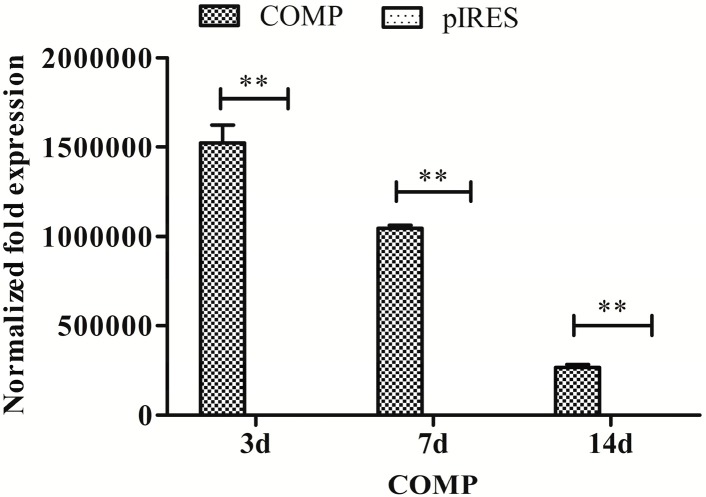
The relative mRNA expression of human cartilage oligomeric matrix protein (*COMP*) was analyzed by real-time PCR at different time points. The value was normalized to GAPDH (*n* = 3, ** *p* < 0.01).

Real-time PCR assay was performed to confirm the expression level of COMP in MSCs using glyceraldehyde-3-phosphate dehydrogenase (GAPDH) as a reference gene for normalization. In this study, plasmid encoding recombinant human COMP was used and specific primers for human COMP was chosen for the real-time PCR. As shown in Figure 6, cells contacted with HA/COMP films obtained significantly higher (*p* < 0.01) expression of COMP than HA/pIRES films for more than 14 days. Although a gradual reduction of expression was observed, high expression of COMP lasted for more than 14 days.

### 2.4. Osteogenic and Chondrogenic Gene Expression in Mesenchymal Stem Cells (MSCs)

In order to investigate MSCs differentiation at the gene level, real-time PCR was performed to detect the mRNA expression level of related genes at day 3, 7 and 14. In this study, we selected collagen type I alpha 1 (Col1α1), alkaline phosphatase (ALP), runt related transcription factor 2 (RUNX2), bone morphogenetic protein 2 (BMP2), osteopontin (OPN), bone gla protein (BGP) as osteogenic markers and collagen type II alpha 1 (Col2α1), aggrecan (ACAN), sry-related high-mobilitygroup box 9 (SOX9), collagen type X alpha 1 (Col10α1) as chondrogenic markers.

**Figure 7:**
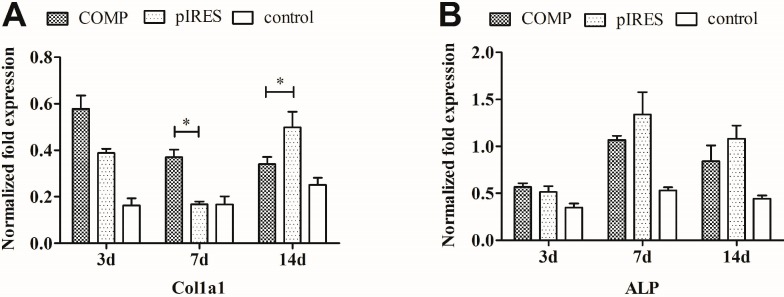

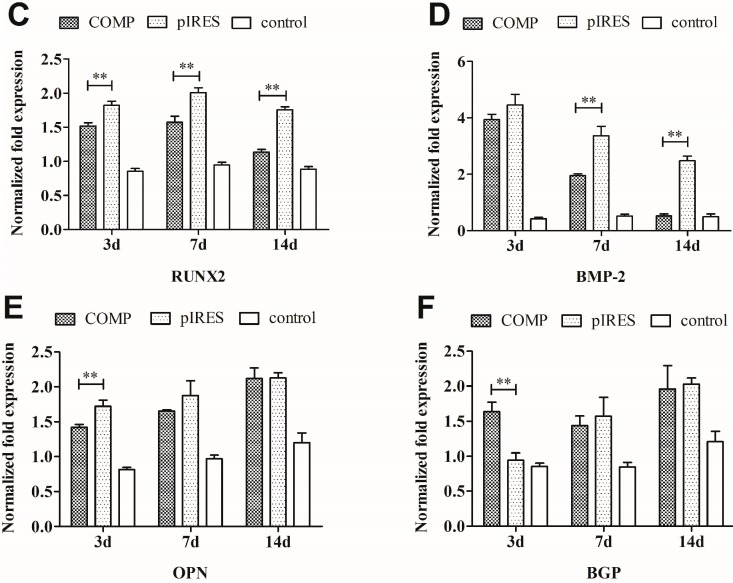
The relative mRNA expression of (**A**) collagen type I alpha 1 (*Col1*α*1*); (**B**) alkaline phosphatase (*ALP*); (**C**) runt related transcription factor 2 (*RUNX2*); (**D**) bone morphogenetic protein 2 (*BMP2*); (**E**) osteopontin (*OPN*) and (**F**) bone gla protein (*BGP*) were analyzed by real-time PCR. The value was normalized to glyceraldehyde-3-phosphate dehydrogenase (GAPDH) (*n* = 3, * *p* < 0.05, ** *p* < 0.01).

HA/COMP films significantly (*p* < 0.01) down-regulated the mRNA expression of *RUNX2* and BMP2 at most time points except for BMP2 at day 3 compared with HA/pIRES films (Figure 7). The functionalized gene films did not exert any significant effect on mRNA expression of ALP, OPN and BGP except for a decrease of OPN and an increase of BGP at day 3. A trend of down regulation of ALP and OPN by HA/COMP films appeared compared with HA/pIRES films but only the decreased expression of OPN at day 3 achieved statistical difference (*p* < 0.01) (Figure 7B,E). Although the expression level of Col1α1 gene in MSCs cultured with HA/COMP films was enhanced at the early stage of osteogenic differentiation (day 3 and 7), a suppression was found after 14 days of culture (Figure 7A). The expression level of BGP in MSCs cultured with HA/COMP films were elevated at the early stage and then arrived at the same level at the late stage compared with HA/pIRES films (Figure 7F).

**Figure 8:**
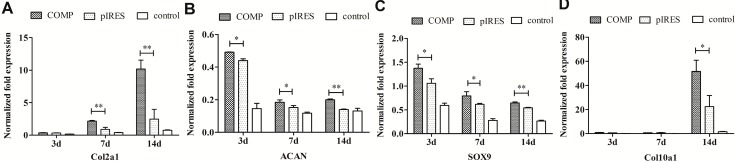
The relative mRNA expression of (**A**) collagen type II alpha 1 (*Col2*α*1*); (**B**) aggrecan (*ACAN*); (**C**) sry-related high-mobilitygroup box 9 (*SOX9*) and (**D**) collagen type X alpha 1 (*Col10*α*1*) were analyzed by real-time PCR. The value was normalized to GAPDH (*n* = 3,* *p* < 0.05, ** *p* < 0.01).

An opposite trend occurred when chondrogenic differentiation assay was performed (Figure 8). A consistent up-regulation of mRNA expression levels of *Col2*α*1*, *ACAN* and *SOX9* for HA/COMP films were observed except for Col2α1 at day 3. Also the expression of *Col2*α*1* increased gradually over time. However, *Col10*α*1* mRNA expression was only up-regulated in MSCs incubated with HA/COMP films at the late stage of chondrogenic differentiation (day 14).

### 2.5. Cell Staining

To further investigate MSCs differentiation cultured with different multilayered films, cell staining was performed for osteogenic differentiation and chondrogenic differentiation.

**Figure 9:**
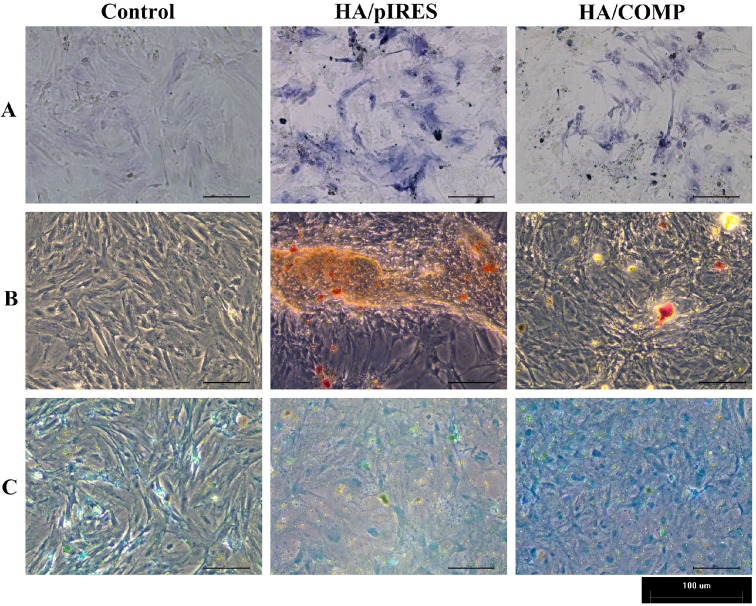
Cell staining of mesenchymal stem cells (MSCs) cultured with TCPs, HA/pIRES films and HA/COMP films. (**A**) alkaline phosphatase (ALP) staining of MSCs after osteogenic induction for 7 days; (**B**) Alizarin red S staining of MSCs after osteogenic induction for 14 days; (**C**) Alcian blue staining of MSCs after chondrogenic induction for 21 days.

Osteogenic differentiation was assessed by alkaline phosphatase (ALP) staining and alizarin red S staining. ALP activity was an early marker of osteogenesis. Also alizarin red S stained the calcium precipitation when mineralized matrix nodules were formed. ALP staining was conducted on day 7. In conformity to the gene expression levels above, both the intensity and the area of ALP staining decreased when MSCs were cultured with HA/COMP films compared with HA/pIRES films, which signified a lower ALP activity (Figure 9A). Alizarin red S staining was conducted at day 14 since calcium precipitation was formed at the late stage of osteogenesis differentiation. Similarly, alizarin red S staining of MSCs cultured with HA/COMP films was weakened according to the fewer number of mineralized nodules (Figure 9B).

Chondrogenic differentiation was assessed by alcian blue staining for the proteoglycans at day 21. In contrast with osteogenic staining, enhanced alcian blue staining of MSCs cultured with HA/COMP films indicated the accumulation of more proteoglycans. Also it agreed with the up regulation of chondrogenic gene expression levels (Figure 9C).

### 2.6. Discussion

Biological coatings have been applied to acquire ideal surface properties of medical devices in different situations. Immobilization of bioactive agents [5,26,27,28,29] and tuning mechanical properties of substrates [10,15,30,31,32] can efficiently direct cell behavior and fate. Deposit of gene components to modify substrates, resulting in a long-term efficiency, is one of the valued strategies [18,19]. As such, genes encoding growth factors such as vascular endothelial growth factor (VEGF) and BMP2 are utilized to promote osteogenic differentiation and bone formation [17,33]. However, gene-functionalized substrates for physiological ligament-bone healing have had lack of attention. As an important non-collagenous protein of the extracellular matrix (ECM) in fibrous cartilage, COMP plays a vital role in cell differentiation and the maintenance of cartilage phenotype [21,22,23,24,34,35].

Therefore, in this study, the plasmid DNA encoding COMP was employed to construct the gene-functionalized films through the LbL technique in consideration of its controlled and sustaining release. Successful construction with rough morphology of the HA/COMP multilayered films was monitored (Figure 1, Figure 2 and Figure 3). The multilayered films were stable in physiological condition and the plasmid could be easily released in enzymatic condition (Figure 4), which agreed with previous studies [36,37]. There were various kinds of enzymes *in vivo* and trypsin was used as a typical one. This indicated that plasmid could be released from the multilayered films *in vivo*. The properties of stability and enzymatic degradation provide the basis of wide application in medical devices. Transfection assay of HEK293T cells demonstrated that the multilayered films could transfect cells and the GFP expression increased with the increasing layer number (Figure 5). This suggested transfection efficiency could be easily regulated by tuning the layer number. It was consistent with other reports [15,37].

To investigate whether the target gene *COMP* could successfully express in MSCs through gene functionalized films, real-time PCR assay of *COMP* was carried out. A higher mRNA expression of COMP was detected in MSCs transfected with HA/COMP films compared with HA/pIRES films (Figure 6). It was reported that the expression level of exogenous gene transfected by liposome reaches a peak after three days culture and could not be sustained for a long time because of the loss of exogenous gene [19,38,39]. The high expression of COMP lasted for more than 14 days, which was much longer than the expression of exogenous gene transfected directly by liposome. This result indicated that the HA/COMP multilayered films could sustain a long-term biological effect for gene transfection.

To further investigate the effect of HA/COMP multilayered films on the osteogenic differentiation of MSCs, real-time PCR of osteoblast-related gene markers was employed. A recent research reported that COMP could bind directly to BMP-2 through the *C*-terminus, inhibit BMP2 receptor binding and block BMP2 osteogenic signaling [24]. BMP2 is known to induce MSCs osteogenic differentiation and stimulate ossification [40]. Meanwhile, RUNX2 is a key transcription factor for osteogenesis and the expression level of RUNX2 could be stimulated by BMP2 [41,42]. In the present study, BMP2 might be blocked by COMP, which could lead to the subsequent down-regulation of RUNX2. All these would contribute to the suppression of osteogenic process. As a result, most of osteoblast-related gene expression levels and osteogenic staining of MSCs cultured with HA/COMP films were restrained compared with HA/pIRES films. It could also be demonstrated by the weaken staining of ALP (Figure 9A). The expression levels of *Col1*α*1* and *BGP* were up-regulated at the early stage and then down-regulated or just flat at the late stage by HA/COMP films (Figure 7A,F). A hypothesis occurred to us that there might be a kind of negative feedback regulation. More research was needed since few studies have focused on this. Weakened alizarin red S staining also indicated the suppression of the mineralization of MSCs cultured with HA/COMP films (Figure 9B). All these suggested that the functionalized gene films could inhibit osteogenic differentiation of MSCs.

Meanwhile, chondrogenic differentiation assay was employed for real-time PCR and alcian blue staining. *Col2*α*1* is one of the most important molecular markers for chondrogenesis. The increasing expression of *Col2*α*1* over time (Figure 8) indicated efficient chondrogenic differentiation and the up-regulated expression of *Col2*α*1* suggested the promotion of chondrogenic differentiation of MSCs by HA/COMP films. The expression levels of SOX9 and ACAN were also up-regulated, which was in accordance with the enhanced alcian blue staining by HA/COMP films (Figure 9C). The expression of *Col10*α*1* was only up regulated at the late stage considering that *Col10*α*1* acted as a late markerof chondrogenic differentiation and it was thought to be an indicator for the maturity and hypertrophy of chondrocytes. COMP is a signaling protein including many domains, such as its *C*-terminals, RGD domains and T3C5 domains [22,23]. Also as a kind of integrin ligand, COMP can recognize and bond integrin α5β1 and start the following signal transduction to direct cell behavior and fate [21]. The results revealed that the functionalized gene films could promote chondrogenic differentiation of MSCs. Altogether, we confirmed that COMP gene multilayers could inhibit osteogenic differentiation and promote chondrogenic differentiation of MSCs *in vitro*.

There are some limitations in our study. As a preliminary study, this study focused on the effect of multilayered COMP films on the differentiation of MSCs *in vitro*. Effects of the multilayered films *in vivo* for ligament-bone healing could not be observed. In a future study, the combination of exogenous MSCs and implants coated with multilayered COMP films for ligament-bone healing *in vivo* will be investigated.

## 3. Experimental Section

### 3.1. Materials

Chitosan (CHI) and hyaluronic acid (HA) were purchased from Sigma Chemical Co. (St. Louis, MO, USA). The pIRES-hrGFP-1a plasmid encoding recombinant human COMP was provided by Sangon Biotech (Shanghai, China). HEK293T cell line was obtained from American type culture collection (ATCC, Shanghai, China). Silicon wafers (20 mm × 20 mm, 1.5 mm thickness; commercially double polishing wafer) and quartz were kindly provided by Guangci Medical Device Co. Ltd. (Hangzhou, China). Glass coverslips (φ = 14 mm) were purchased from NEST (Wuxi, China). Mesenchymal stem cells (MSCs), corresponding stem cell products and cell staining reagents were provided by Cyagen (Guangzhou, China) except for special note. Information of the components of the osteogenic (Catalog NO. RASMX-90021) and chondrogenic (Catalog NO. RASMX-90041) media can be obtained on the Cyagen official website.

### 3.2. DNA Complex Preparation

pIRES-hrGFP-1a (pIRES) or pIRES–hrGFP-1a–COMP (COMP) plasmid DNA complex was prepared using Lipofectamine™ LTX and PLUS reagent (Invitrogen, Carlsbad, CA, USA) according to the manufacturer’s instruction. In brief, 100 µg of plasmid DNA was diluted with Opti-MEM (Life Technologies, Carlsbad, CA, USA) and mixed with 100 µL PLUS reagent and 300 µL LTX reagent to a final volume of 10 mL (LDc solution). Then the mixture was incubated at room temperature for 30 min before fabrication.

### 3.3. Pretreatment of Substrates

Silicon wafers, quartz substrates and glass coverslips chosen for the fabrication were cleaned following the procedure for multilayered films fabrication as described by Wang, X.F. *et al.* [43]. Namely, the substrate was cleaned sequentially in methanol, ethanol, methanol and water, immersed in a Piranha solution (7:3 (vol. %), 98% H_2_SO_4_:30% H_2_O_2_) for 10 min, and then 1:1:5 (vol. %), 30% H_2_O_2_:25% NH_3_:H_2_O at 60 °C for 30 min, finally washed with deionized water and dried at room temperature before use. All these chemical reagents were provided by Sinopharm Chemical Reagent Co., Ltd. (Shanghai, China).

### 3.4. Fabrication of Multilayered Films

**Scheme 1:**
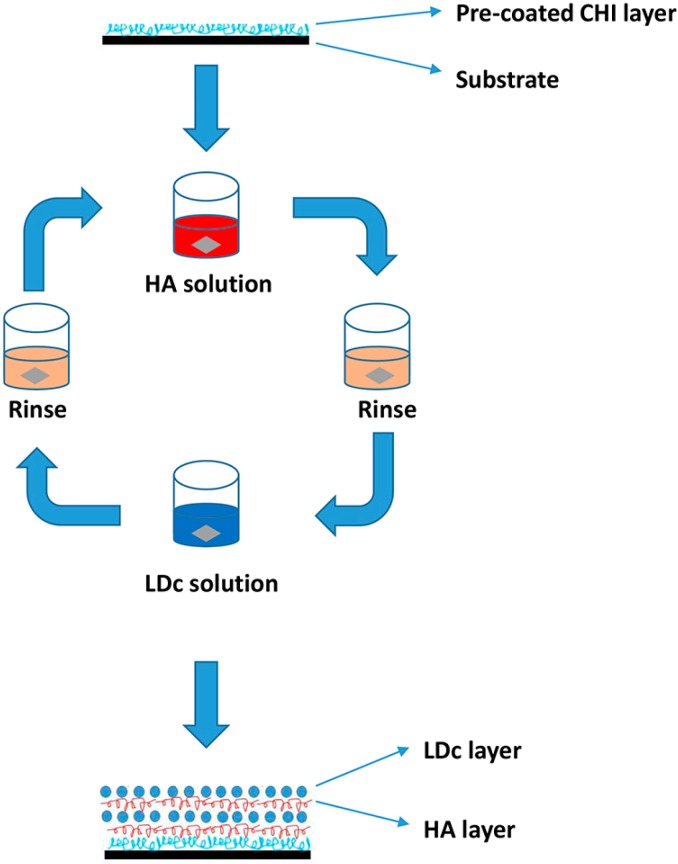
Layer-by-layer assembly of (HA/DNA)_n_ films. Substrate was first coated with Chitosan (CHI) and submerged in HA solution. Following a rinse in Opti-MEM twice, the substrate was then immersed in the lipid-DNA complex (LDc) solution. Another rinse was conducted before the next round.

CHI solution (5 mg/mL) was prepared with 2% (vol. %) acetic acid and HA was dissolved in distilled water with a concentration of 0.5 mg/mL. The substrates were originally dipped in CHI solution for 30 min to form a precursor layer with stable positive charge to initiate the following procedure. Then the (HA/DNA)_n_ (*n* is the number of bilayer) multilayered films were fabricated by immersing substrates in HA solution for 10 min. After being washed twice with Opti-MEM for 1 min, substrates were deposited in LDc solution for 10 min and washed again. These procedures were repeated until the desired multilayered films were fabricated (Scheme 1). (HA/COMP)_n_ films were used in the follow-up characterization and cell experiments. Also (HA/pIRES)_n_ films were chosen as negative control in cell experiments.

### 3.5. Characterization of the Multilayered Films

The build-up process of films was monitored through contact angle measurement by a video-based contact angle measuring device (OCA 20, Dataphysics, Stuttgart, Germany). The images of water drops on the surface were captured and analyzed following the instructions. Three samples in each group were chosen and three different points in each sample were measured.

The progressive buildup of films was also measured on a UV–vis spectrophotometer (Bio-Rad, Smart Spec™ Plus, Hercules, CA, USA). The UV–vis absorption at 260 nm of multilayered films on quartz slides was detected. Six samples in each group were used.

The morphology of multilayered films on silicon wafers was determined by a scanning electron microscopy (SEM; Hitachi, TM-1000, Tokyo, Japan). Samples were dried at room temperature after fabrication and sputter-coated with gold before detection.

Films with six bilayers of HA/COMP were selected for the stability assay. The multilayered films were exposed to PBS buffer (pH = 7.4, Life Technologies, Carlsbad, CA, USA) at 37 °C. At appropriate time intervals, films were taken out, washed with deionized water, and dried before the measurement of UV–vis absorption at 260 nm. Three samples were chosen as replicates.

Trypsin was chosen as the model protease to investigate the degradation properties of (HA/COMP)_6_ films for the enzymatic degradation assay. The degradation samples were incubated in a trypsin solution (0.1 mg/mL in PBS buffer, pH = 7.4) at 37 °C. The films were taken out of the incubation solution at desired time intervals and washed with PBS buffer before UV–vis absorption detection using a UV–vis spectrophotometer at the wavelength of 260 nm. Also the incubation solution was measured at the same time. Three replicates were used as above.

### 3.6. In Vitro Gene Transfection

To assess the biological activity of the multilayered films, the (HA/COMP)_n_ (*n* = 2 or 6) films were fabricated on glass coverslips and 293T cells were selected for the transfection experiment. The 293T cells were seeded (density 1 × 10^4^ cells/cm^2^) into 24-well plates (TCPs) to which multilayered films had been added in dulbecco’s modified eagle medium (DMEM, Life Technologies, Carlsbad, CA, USA) at 37 °C and 5% CO_2_. Cells in TCPs without multilayered films were selected as control and transfected with Lipofectamine™ LTX and PLUS reagent according to the manufacturer’s instructions if mentioned. After incubation for three days, GFP expression of 293T cells was observed under a fluorescence microscope (Leica, DMI4000B, Solms, Germany). Fluorescence counts of different films were calculated by Image-Pro Plus 6.0 (Media Cybernetics, Rockville, MD, USA). The mean value of five replicates was used as the final result for each group.

### 3.7. Transfection and Differentiation of MSCs

The (HA/COMP)_6_ and (HA/pIRES)_6_ films were chosen in this study for the following cell experiments. MSCs were seeded (initial density 2 × 10^4^ cells/cm^2^) into 24-well plates in MSC growth medium for 4 h adhesion, after which the selected films were gently put on top of the cells. Then cells were cultured for 48 h, followed by the process of MSC differentiation by replacing the growth medium with osteogenic or chondrogenic differentiation medium. Cells were harvested at appropriate time intervals for subsequent analysis. For COMP expression detection, growth medium was sustained till the end of the assay and cells were collected at desired time points for real-time PCR analysis.

### 3.8. RNA Isolation and Real-Time PCR

The total RNA of MSCs harvested at specified time points was extracted using TRIZOL reagent (Invitrogen, Carlsbad, CA, USA) according to the manufacturer’s protocol. Reverse transcription reactions were carried out using Prime Script RT Reagent Kit (Takara, Dalian, China) according to the manufacturer’s instructions. Real-time PCR was performed using SYBR Premix Ex Taq (Takara, Dalian, China) according to the manufacturer’s instructions. Target genes were normalized to GAPDH expression and all sample values were calculated by the 2^−ΔΔ*C*t^ method. Table 1 shows the primer sequences used in this study.

**Table 1:** Primer oligonucleotide sequences in this study.

Gene	Oligonucleotides (5'–3')	Gene Bank ID
*GAPDH*	F: GACATGCCGCCTGGAGAAAC	NM_017008.4
	R: AGCCCAGGATGCCCTTTAGT	
*RUNX2*	F: GCGTCCTATCAGTTCCCAAT	NM_001278483.1
	R: CAGCGTCAACACCATCATTC	
*OPN*	F: CTTGGCTTACGGACTGAGG	NM_012881
	R: GCAACTGGGATGACCTTGAT	
*BMP2*	F: TGAGGATTAGCAGGTCTTTGC	NM_017178.1
	R: TCTCGTTTGTGGAGTGGATG	
*BGP*	F: CAAGTCCCACACAGCAACTC	NM_013414.1
	R: CCAGGTCAGAGAGGCAGAAT	
*COMP*	F: CCCAACTCAGACCAGAAGGA	NM_000095.2
	R: GTCACAAGCATCTCCCACAA	
*SOX9*	F: GACGTGCAAGCTGGGAAAGT	XM_001081628.3
	R: CGGCAGGTATTGGTCAAACTC	
*Col2*α*1*	F: CGCCACGGTCCTACAATGTC	NM_012929.1
	R: GTCACCTCTGGGTCCTTGTTCAC	
*ACAN*	F: TGGCATTGAGGACAGCGAAG	NM_022190.1
	R: TCCAGTGTGTAGCGTGTGGAAATAG	
*Col10*α*1*	F: GGGATGCCTCTTGTCAGTGC	XM_001053056.4
	R: ATCTTGGGTCATAGTGCTGCTG	
*Col1*α*1*	F: GACATGTTCAGCTTTGTGGACCTC	NM_053304.1
	R: GGGACCCTTAGGCCATTGTGTA	

### 3.9. Cell Staining

The cells cultured in osteogenic differentiation medium for seven days were stained using BCIP/NBT Alkaline Phosphatase Color Development Kit (Boster, Wuhan, China) for alkaline phosphatase (ALP) staining according to the protocol.

On day 14 after osteogenic differentiation, alizarin red S staining for calcium precipitation was performed as described before [44,45]. Briefly, cells were fixed with 4% formaldehyde solution for 30 min, followed by washing twice with PBS. Then cells were stained with alizarin red S for 5 min. After rinsing with PBS twice again, the staining of mineralized nodules was observed under a light microscope (Leica, DMIL, Solms, Germany).

On day 21 after chondrogenic differentiation, alcian blue staining for proteoglycans synthesized by chondrocytes was performed. Cells were washed with PBS, fixed with 4% formaldehyde solution (Sinopharm Chemical Reagent Co., Ltd., Shanghai, China) for 30 min and washed again with PBS. Cells were stained with alcian blue solution (Cyagen, Guangzhou, China) for 30 min and washed with distilled water (Life Technologies, Carlsbad, CA, USA). Then cells were observed under a light microscope.

### 3.10. Statistical Analysis

The results were expressed as mean ± standard deviation (SD). An unpaired Student’s *t*-test was performed to evaluate the significance of the observed differences between the study groups and a value of *p* < 0.05 was considered to be statistically significant.

## 4. Conclusions

In summary, we developed an efficient COMP gene delivery system to regulate cell differentiation in this study. COMP plasmid DNA was successfully incorporated into multilayered films with HA via the LbL technique. Multilayered films, which were demonstrated to possess the property to transfect MSCs and induce continuous expression of COMP for at least two weeks, could inhibit osteogenic differentiation and promote chondrogenic differentiation of MSCs *in vitro*. The combination of surface modification and functional gene therapy provides a promising strategy for potential applications in the field of medical devices for physiological ligament-bone healing.
